# Acute Kidney Injury Following Duloxetine (Cymbalta) Therapy in a 43-Year-Old Female: A Case Report

**DOI:** 10.7759/cureus.101380

**Published:** 2026-01-12

**Authors:** Akhtar Purvez, Sana Khan, Mudhasir Bashir

**Affiliations:** 1 Clinical Research, Momentum Medical Research, Charlottesville, USA; 2 Clinical Sciences, Lincoln Memorial University DeBusk College of Osteopathic Medicine, Harrogate, USA; 3 Nephrology, University of Virginia, Charlottesville, USA; 4 Psychiatry and Behavioral Sciences, University of Virginia, Charlottesville, USA

**Keywords:** acute kidney injury, adverse drug reaction (adr), drug-induced nephrotoxicity, duloxetine, serotonin-norepinephrine reuptake inhibitor (snri)

## Abstract

Duloxetine is a commonly prescribed serotonin-norepinephrine reuptake inhibitor (SNRI) used to treat depression, anxiety disorders, and chronic pain syndromes. Renal adverse effects are considered rare. We present the case of a 43-year-old female who developed acute kidney injury (AKI) eight weeks after initiating duloxetine and escalating the dose from 30 mg to 60 mg daily, necessitating hospitalization. The patient presented with mental lethargy, disorientation, and generalized weakness and was found to have marked azotemia with a significant reduction in estimated glomerular filtration rate. Her condition improved following intravenous hydration and discontinuation of all medications, with gradual normalization of renal function over approximately six weeks. Notably, the patient had no history of dehydration, gastrointestinal losses, or reduced oral intake before presentation. Hemoglobin and hematocrit levels remained normal throughout the course of AKI. The close temporal association with duloxetine initiation and recovery following drug cessation supports a probable causal relationship. This report highlights the importance of vigilance for potential renal adverse effects associated with SNRIs.

## Introduction

Acute kidney injury (AKI) is a common and clinically significant condition associated with increased morbidity, hospitalization, and long-term risk of chronic kidney disease [1]. Drug-induced nephrotoxicity is a major and potentially preventable cause of AKI, including acute interstitial nephritis induced by medications [2,3]. Duloxetine is a serotonin-norepinephrine reuptake inhibitor (SNRI) approved for the treatment of major depressive disorder, generalized anxiety disorder, diabetic peripheral neuropathic pain, fibromyalgia, and chronic musculoskeletal pain [4]. Because duloxetine metabolites are primarily eliminated by the kidneys, caution is advised in patients with renal impairment, and use is not recommended in advanced renal failure [5,6]. Despite its widespread use, duloxetine-associated AKI remains poorly described, with only isolated case reports in the literature [7]. We present a case of severe AKI temporally associated with duloxetine initiation and dose escalation, with recovery following drug discontinuation and supportive care.

## Case presentation

A 43-year-old female presented to the emergency department with progressive lethargy, disorientation, and generalized weakness. She had a history of chronic pain related to complex regional pain syndrome (CRPS) type I involving the right lower extremity. Pain severity ranged from 5 to 8 of 10 during exacerbations. She had been managed with intermittent lumbar sympathetic blocks using local anesthetics, which consistently provided moderate pain relief lasting several months. More recently, she experienced worsening symptom severity and increased functional limitations. She had failed multiple medication classes commonly used for neuropathic pain, including antiepileptic agents such as gabapentin, pregabalin, and topiramate, as well as topical therapies such as lidocaine patches. Eight weeks before presentation, duloxetine had been initiated at 30 mg daily for chronic neuropathic pain. She had initially tolerated therapy without reported adverse effects. After four weeks, the dose had been increased to 60 mg daily due to a partial response. After four more weeks, she had started noticing the current symptoms. Her medical history was also notable for hypertension, well-controlled on losartan 50 mg daily for more than five years. She had no known chronic kidney disease, diabetes mellitus, autoimmune disease, recent infection, or contrast exposure. Notably, there was no history of dehydration, vomiting, diarrhea, excessive diuretic use, or reduced oral intake before presentation. She denied any use of nonsteroidal anti-inflammatory drugs, herbal supplements, or other nephrotoxic agents. Table 1 presents the admission renal parameters. During the course of AKI, hemoglobin and hematocrit stayed within normal ranges. The urinalysis was normal, and the renal imaging showed no signs of blockage.

**Table 1:** Admission laboratory values demonstrating AKI AKI: acute kidney injury; BUN: blood urea nitrogen; eGFR: estimated glomerular filtration rate

Parameter	Value	Units	Reference range
BUN	83	mg/dL	8–26
Serum creatinine	3.0	mg/dL	0.7–1.3
eGFR	20	mL/min/1.73 m²	>60

**Figure 1:**
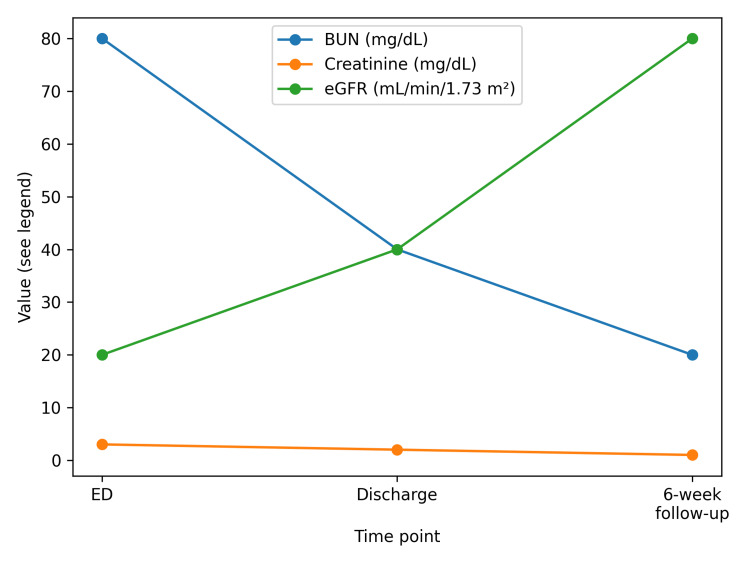
Renal parameter progression ED: emergency department; BUN: blood urea nitrogen; eGFR: estimated glomerular filtration rate

The patient was admitted for management of AKI. All outpatient medications, including duloxetine and losartan, were discontinued. She received intravenous isotonic fluids and close monitoring. Her mental status improved as renal parameters stabilized. She was discharged after three days with blood urea nitrogen (BUN) 41 mg/dL, creatinine 2.1 mg/dL, and estimated glomerular filtration rate (eGFR) approximately 40 mL/min/1.73 m². Over six weeks, renal function returned to baseline. Duloxetine was not restarted. Losartan was later reintroduced without recurrence of renal dysfunction. Table 2 shows the details of previous medication exposure. Table 3 depicts the clinical timeline, and Table 4 shows the progression of renal parameters. Figure 1 illustrates these parameters in a graphic representation.

**Table 2:** Medication exposure irior to and during AKI AKI: acute kidney injury; NSAIDs: nonsteroidal anti-inflammatory drugs

Medication	Dose	Duration	Status
Duloxetine	30 mg → 60 mg daily	8 weeks total	Discontinued
Losartan	50 mg daily	>5 years	Temporarily stopped
NSAIDs	—	None	Not used
Herbal supplements	—	None	Not used
Contrast exposure	—	None	None

**Table 3:** Clinical timeline AKI: acute kidney injury; IV: intravenous

Time point	Event
Week 0	Duloxetine initiated (30 mg)
Week 4	Dose increased to 60 mg
Week 8	Neurologic symptoms and AKI
Hospital days 1–3	IV fluids, medication cessation
Week 6	Renal recovery complete

**Table 4:** Progression of renal parameters ED: emergency department; BUN: blood urea nitrogen; eGFR: estimated glomerular filtration rate

Parameter	Reference range	ED Presentation	Discharge	6-week follow-up
BUN (mg/dL)	8–26	83	41	Normal
Creatinine (mg/dL)	0.7–1.3	3.0	2.1	Normal
eGFR (mL/min/1.73 m²)	>60	20	40	>60

## Discussion

Duloxetine is a commonly prescribed medication for multiple indications. It is usually well-tolerated with mild and transient side effects, including headaches, decreased appetite, and sweating. Serious side effects are rare. The FDA prescribing information does not list AKI as a common or uncommon adverse effect. This case demonstrates a probable association between duloxetine therapy and AKI. The causal relationship is substantiated by the temporal correlation between the commencement and dose escalation of duloxetine, the emergence of symptoms, and the subsequent recovery following drug cessation, with the Naranjo score of 6 further validating this classification [8]. Table 5 describes this score as applicable to this patient.

**Table 5:** Naranjo score

Question	Response	Score
Temporal relationship	Yes	+2
Improvement on drug withdrawal	Yes	+1
Dose-response relationship	Yes	+1
Alternative causes	Less likely	+1
Objective evidence	Yes	+1
Total score		6 (probable)

Angiotensin receptor blockers such as losartan can cause hemodynamically mediated reductions in glomerular filtration rate, particularly in states of dehydration or reduced effective circulating volume [9,10]. In this case, there was no history of dehydration or gastrointestinal losses, hemoglobin and hematocrit remained within normal limits, and the patient had tolerated losartan for more than five years, making it an unlikely primary driver.

Potential mechanisms for duloxetine-associated AKI remain speculative and may include idiosyncratic tubular toxicity or drug-induced acute interstitial nephritis [2,3]. Published data are sparse, with one report describing urinary retention complicated by AKI following duloxetine exposure [7]. Previous reports have emphasized the value of careful temporal documentation and conservative management in drug-associated AKI, including the one illustrated in our report of AKI following intravenous iron sucrose [11-15].

## Conclusions

This report highlights a rare but clinically significant episode of AKI temporally associated with duloxetine therapy initiated for the management of chronic pain related to CRPS type I. A probable drug-related adverse effect is supported by the close temporal relationship between drug initiation and dose escalation, objective biochemical evidence of renal injury, absence of dehydration or alternative nephrotoxic exposures, and gradual recovery following drug discontinuation. Clinicians should remain vigilant for potential renal adverse effects when initiating or escalating SNRIs, even in patients without known kidney disease or traditional risk factors for AKI. Early recognition of symptoms, prompt laboratory evaluation, and timely withdrawal of the suspected agent may allow full renal recovery and prevent progression to persistent kidney dysfunction. As the use of SNRIs continues to expand across psychiatric and chronic pain indications, awareness of rare but serious renal complications is essential for safe prescribing and effective pharmacovigilance.

## References

[1] (2012). KDIGO Clinical Practice Guideline for acute kidney injury. Kidney Int Suppl.

[2] Perazella MA, Markowitz GS (2010). Drug-induced acute interstitial nephritis. Nat Rev Nephrol.

[3] Perazella MA (2009). Renal vulnerability to drug toxicity. Clin J Am Soc Nephrol.

[4] (2026). CYMBALTA (duloxetine hydrochloride) prescribing information. Eli Lilly and Company. https://uspl.lilly.com/cymbalta/cymbalta.html#pi.

[5] Lantz RJ, Gillespie TA, Rash TJ, Kuo F, Skinner M, Kuan HY, Knadler MP (2003). Metabolism, excretion, and pharmacokinetics of duloxetine in healthy human subjects. Drug Metab Dispos.

[6] Lobo ED, Heathman M, Kuan HY (2010). Effects of varying degrees of renal impairment on the pharmacokinetics of duloxetine: analysis of a single-dose phase I study and pooled steady-state data from phase II/III trials. Clin Pharmacokinet.

[7] Aktürk Esen S, Gül CB, Kahvecioğlu S, Aktaş N, Esen İ (2019). Acute urinary retention and acute kidney injury after duloxetine treatment: a rare case report. J Clin Psychopharmacol.

[8] Naranjo CA, Busto U, Sellers EM (1981). A method for estimating the probability of adverse drug reactions. Clin Pharmacol Ther.

[9] Holtkamp FA, de Zeeuw D, Thomas MC (2011). An acute fall in estimated glomerular filtration rate during treatment with losartan predicts a slower decrease in long-term renal function. Kidney Int.

[10] Alshahrani S (2023). Renin-angiotensin-aldosterone pathway modulators in chronic kidney disease: a comparative review. Front Pharmacol.

[11] Purvez A, Khan S, Abdel-Rahman E, Malik N, Bashir M (2025). Acute kidney injury following intravenous iron sucrose in a 67-year-old male: a case report. Cureus.

[12] Bailie GR, Clark JA, Lane CE, Lane PL (2005). Hypersensitivity reactions and deaths associated with intravenous iron preparations. Nephrol Dial Transplant.

[13] Auerbach M, Macdougall IC (2014). Safety of intravenous iron formulations: facts and folklore. Blood Transfus.

[14] Zager RA (2006). Parenteral iron compounds: potent oxidants but mainstays of anemia management in chronic renal disease. Clin J Am Soc Nephrol.

[15] Clark BA, Osadchuk L, John J, Culver T, Marcus R (2016). Effect of intravenous iron on outcomes of acute kidney injury. Transfusion.

